# Maternal Vitamin A deficiency during pregnancy and lactation induced damaged intestinal structure and intestinal flora homeostasis in offspring mice

**DOI:** 10.1002/fsn3.3332

**Published:** 2023-04-12

**Authors:** Junming Zhou, Bo Sun, Minli Li, Haoyu Xu, Ying Feng, Xiaowei Wu, Meixia Guo, Xiaomin Wang

**Affiliations:** ^1^ Department of Cadre Gastroenterology, Jinling Hospital Medical School of Nanjing University Nanjing China; ^2^ Fifth Station Outpatient Department of Jinling Hospital Medical School of Nanjing University Nanjing China

**Keywords:** intestinal barrier, intestinal inflammatory, intestinal microbiota, Vitamin A

## Abstract

The small intestine serves as the first channel of dietary Vitamin A (VA) and the unique organ of VA absorption and metabolism. However, there have not been extensive investigations on the exact mechanisms within VA‐related changes in intestinal metabolic disorders. This research is designed to analyze whether and how VA affects intestinal metabolic phenotypes. Male C57BL/6 mice after weaning were randomly fed a VA control diet (VAC) or a VA‐deficient diet (VAD) during the entire pregnancy and lactation process. After a total of 11 weeks, cohorts of VA deprived were next fed to a VA control diet (VAD‐C) for another 8 weeks. The concentration of retinol was measured by a high‐performance liquid chromatography system. The 16S gene sequencing was used to evaluate the intestinal microbiota changes. Through the use of histological staining, western blots, quantitative PCR, and enzyme‐linked immunosorbent assays, the intestinal morphology, inflammatory factors, and intestinal permeability were all evaluated. Following the decrease of the tissue VA levels, VAD mice show a decrease in tissue VA levels, community differences, and the richness and diversity of intestinal microbiota. VAD diet‐driven changes occur in intestinal microbiota, accompanied by a higher mRNA expression of intestinal inflammatory cytokines and an increase in intestinal permeability. As dietary VA is reintroduced into VAD diet‐fed mice, the tissue VA levels, inflammatory response, and intestinal homeostasis profiles are all restored, which are similar to those found after the occurrence of VA‐controlled changes within intestinal microbiota. VA deficiency caused the imbalance of intestinal metabolic phenotypes through a mechanism involving changes in intestinal microbiota. It is thought that intestinal microbiota metabolic influences represent a new salient and additional mechanism, which can be used as a new method to achieve the onset and treatment of the effect of VAD on intestinal homeostasis impairment.

## Graphical abstract

Vitamin A deficiency causes the imbalance of intestinal metabolic phenotypes through a mechanism involving changes in intestinal microbiota. Akkermansia may be used as a potential therapeutic target for the treatment of the effect of VA deficiency on intestinal homeostasis impairment.

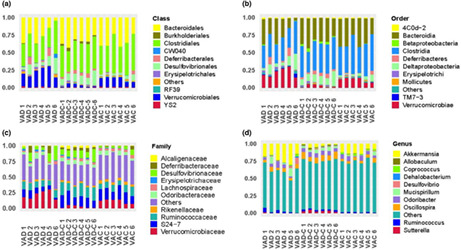

## 1 INTRODUCTION

Vitamin A (VA) and its derivatives, which are in different retinoid forms, play an essential role in keeping tissue homeostasis, organ differentiation, and cell apoptosis (Ghyselinck & Duester, 2019; Zhou et al., 2021). Their deficiency, which is identified as VA deficiency (VAD), was regarded as one of the three major trace element deficiency diseases in developing countries (Hodge & Taylor, 2021; Surman et al., 2020). In a prospective study, a conclusion is that serum retinol concentrations were inversely related to the increased risk of gastrointestinal and respiratory morbidity, and diarrhea with vomiting (Thornton et al., 2014). In previous in vitro studies, it is also reported that all‐trans retinoic acid (RA) accelerates the attenuation of intestinal adaptability (Hong et al., 2021), enhances differentiation and permeability of intestinal cells (Yamada & Kanda, 2019), and increases the stemness of intestinal stem cells (Wang et al., 2020). However, it is worth noting that there is still an unknown influence of the dietary VA itself on keeping both intestinal homeostasis and functions.

Recently, special attention is on the role of gut microbiota in the regulation of keeping intestinal homeostasis within inflammatory bowel diseases and some other diseases (Di Pierro, 2021; Sommer et al., 2017). Referred as a “hidden organ”, it is found that intestinal microbiota exerts some roles in the regulation of gut homeostasis by offering signals to sentinel and other regulatory cells in the gut to secrete factors driving host responses to various stimuli, which has a range from nutrient influx to inflammation (Cani et al., 2009; Fredborg et al., 2012). Looking at the recent progress, Brauer‐Nikonow and Zimmermann (2022) pointed out that the balance of intestinal retinoic acid concentrations plays a necessary role in keeping immune homeostasis within the gut. Although supplementation and/or biofortification with VA are common to combat VAD, the influence of VA on intestinal health during VAD has not been fully clarified, especially regarding intestinal dysbiosis.

We proposed a hypothesis that dietary VA is likely to also have beneficial impacts through perturbing the complicated and large microbial community residing within the gastrointestinal tract (intestinal microbiome). In view of this, we adopted a diet‐induced model of VAD that shows fecal dysbiosis and intestinal dysfunctions linked to their VAD status. Taking into account that VA‐driven changes in intestinal microbiota have an impact on intestinal dysfunction, a new therapeutic method for VAD‐driven intestinal impairment via intestinal microbiota modulation can be explored.

## 2 MATERIALS AND METHODS

### 2.1 Animals and diet

The model mice were carried out following a previously shown method with a certain amount of modifications (Zhou et al., 2020). Female C57BL/6 pregnant mice were randomly divided into two groups, which were the VA‐deficient (VAD) group (VA <120 IU/kg) and the VA control (VAC) group (4000 IU/kg VA) (*n* = 6 per group). In the VAC group, pregnant mice and selected offspring male mice were fed with the VAC diet continuously to 22 weeks old. VAD group is that pregnant mice and selected offspring male mice were fed with VAD diet continuously to 22 weeks old. In addition, the VAD‐C group is defined as that after feeding with the VAD diet to 14 weeks old, selected offspring male mice were re‐fed with the VAC diet to 22 weeks old. All mice were housed in a constant temperature (12‐h day/light cycle) room with food and water available ad libitum. At indicated times, mice were sacrificed and samples were collected for further use. Each of the animal experiments was approved by the Animal experimentation ethical committee of Nanjing Medical University. Also, each of the experimental procedures was conducted in accordance with the National Institutes of Health Guide for the Care and Use of Laboratory Animals (NIH Publication No. 8023, revised 1978).

### 2.2 Retinoid determination

By the previously described method, the retinol concentrations within serum and tissues were identified at the wavelength of 340 nm through the application of a high‐performance liquid chromatography system (HPLC, Waters Millennium, Waters) as retinol for standard (Zhou et al., 2020). For serum detection, the serum was deproteinized through the use of alcohol and then extracted with hexane for further experiments. As for the tissue detection, the frozen tissues were homogenized within phosphate balanced solution (PBS) (tissue weight (g): PBS (ml) volume = 1:1) in dark. Next, sample retinoid was extracted through the organic solution, and detected by using the gradient elution of the mobile phase within a liquid chromatograph that is equipped with a 315‐nm ultraviolet photodiode array detector. The tissue retinol levels were normalized to milligram of the tissue weight.

### 2.3 Quantitative PCR (q‐PCR)

The total RNA was isolated and extracted from tissue via the RNeasy kits (Vazyme), and was used to synthesize reverse transcribed through the use of the PrimeScript RT Reagent Kit (TaKaRa Bio). The GenBank database was used to design the specific mRNA primers for target gene amplification (Table 1). qPCR was performed through the use of the SYBR Green PCR Master Mix (Takara Bio) within the Step One Real‐Time PCR System (Applied Biosystems) under the standard with β‐actin as an endogenous control. Besides, the ΔCt method was used to quantify the relative mRNA levels.

**TABLE 1:** Sequences of primers used for quantitative PCR.

Gene	Primer sequence (5′‐3′)	
RARα	F: GCAAAAGGAAGTGCTCGGTG	R: CACTCTCGTACATCTCGCCC
RARβ	F: GTGCCCATACTGGTGTCTCC	R: CTTAGGGTTCTGGGGCAGTG
RXRα	F: ACCCACTCATTGACTCCCCT	R: AGCTCAGGGTGCTGATAGGA
RXRβ	F: TGGGAGCCATCTTTGATCGG	R: ATAGGTCTCCAGTGAGGCGT
CRBP2	F: TCCTTCACAGTCACCGAACG	R: CTTGCGGGTGGCAAAATCAA
CYP26a1	F: CTGGGACCTGTACTGTGTGAG	R: CTGCTGACTTCCTCAGCGAT
β‐actin	F: AGGGAAATCGTGCGTGACAT	R: CGCAGCTCAGTAACAGTCCG

### 2.4 PCR amplification

Following the manufacturer's instructions, Metagenomic DNA from the cecal content of chosen male mice (six per group) was extracted through the application of the QIAamp® DNA Stool Mini Kit (Qiagen). One percent agarose gel electrophoresis was used to quantify DNA. A set of primers was used to amplify the V4–V5 hypervariable region of the bacterial 16S ribosomal RNA gene. In addition, the specific primers included the 441F (5′‐CCTACGGGNGGCWGCAG‐3′) and the 825R (5′‐GACTACHVGGGTATCTAATCC‐3′). PCR reactions were conducted in a 50‐μL volume system that contains 12.5 ng DNA, 0.1 μM of primers, and a mixture of the KAPA HiFi Hot start Ready Mix. Under the conditions below, the amplification program was carried out: 94°C for 3 min, followed by 18 cycles of 94°C for 30 s, 55°C for 30 s, and 72°C for 30 s, respectively, with a final extension at 72°C for 5 min. Following the manufacturers' instructions, the Qubit quantification system (Thermo Scientific) was used to check and quantify the amplicons.

### 2.5 Illumina MiSeq System sequencing

The purified amplicons were pooled employing the AxyPrep DNA Gel Extraction Kit (Axygen Biosciences) following the manufacturer's instructions, and were quantified through the use of the QuantiFluorTM‐ST (Promega). The Qubit quantification system was used to decide the pooled libraries concentration. Then, the amplicon sequencing was carried out upon the Illumina MiSeq System (Illumina Inc.) that was performed by the Majorbio Bio‐Pharm Technology Co., Ltd. A composite of dual‐index reads was applied to carry out the automated cluster generation and paired‐end sequencing.

### 2.6 Informatic sequence analysis

Operational taxonomic units (OTUs) were explored through the use of phylogenies within the Quantitative Insights into Microbial Ecology (QIIME) software (Version 1.9.0) (Kuczynski et al., 2011). In QIIME, the command split library database was used for taxonomy assignment. The beta‐diversity was calculated by unweighted UniFrac distances and visualized with principal coordinate analysis (PCoA) to compare differences among microbial community profiles. Variable importance in projection (VIP) with a cut‐off value of 1.0 was employed to identify OTUs that made the largest contribution to discriminating microbial communities among groups. The partial least squares discriminant analysis (PLS‐DA) was estimated using the mixOmics package among three groups. Heatmap analysis and community structure component diagrams were generated in accordance with the taxonomic information of each sample by Metastats according to a previous study (White et al., 2009).

### 2.7 Histology

Following standard methods for histological examination, paraffin‐embedded intestinal tissue sections (4 μm) were dehydrated. Sections were fixed with 4% formaldehyde and were then stained with hematoxylin and eosin (H&E). In addition, all of the sections were explored through the Image Pro Plus 4.5.1 software (Media Cybernetics).

### 2.8 Intestinal inflammatory response system measurement

Following the manufacturer's instructions, the degrees of tumor necrosis factor‐α (TNF‐α), interleukin‐6 (IL‐6), and interleukin‐8 (IL‐8) in intestinal tissues among the groups were prepared and detected by using the specific TNF‐α, IL‐6, and IL‐8 measurement kit (both from JianchengBio).

### 2.9 Statistical analysis

Data represent at least three independent experiments and are expressed as mean ± standard error. The differences were assessed through the separate use of the Student's t‐test and analysis of variance (ANOVA). Each statistical analysis was carried out through the use of the Prism v.6.0 software (GraphPad). In the meantime, the significance was assumed to be *p* < .05.

## 3 RESULTS

### 3.1 VA deficiency impairs tissue retinol levels and the way of VA metabolism

**FIGURE 1:**
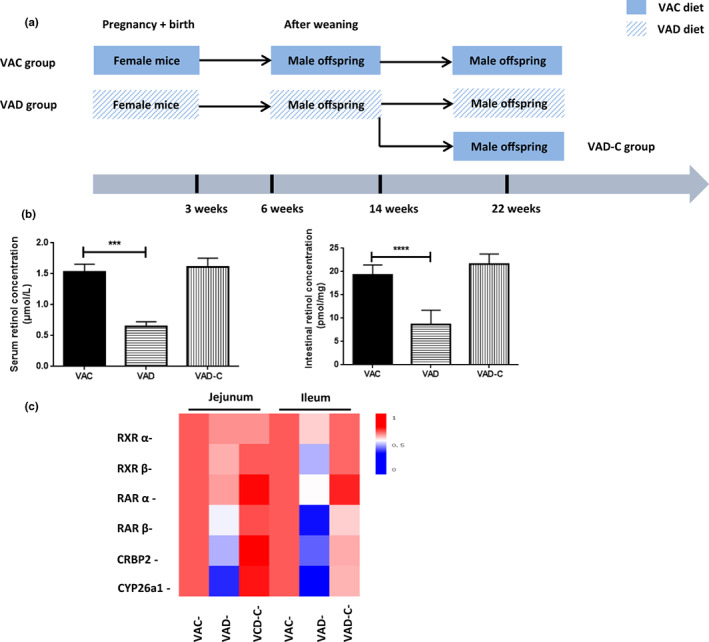
VAD decreased tissue retinol levels and mRNA expression of the VA metabolic signaling pathway. (a) The following flow of this study. (b) VA levels in the serum and intestines of mice from VAC, VAD, and VAD‐C groups. (c) Heatmap of PCR measurements of relative pancreatic/intestinal mRNA levels involved in VA metabolic signaling of mice from VAC, VAD, and VAD‐C groups. ***p < .001, ****p < .0001 in post hoc comparisons among groups after one‐way ANOVA analysis.

Following the timeline shown in Figure 1a, our diet‐induced VAD model was performed. Lower levels of serum retinol were detected in VAD groups compared with VAC groups (0.62 vs. 1.52 μmol/L, *p* < .001). However, compared to those within the VAC group, intestinal VA (retinol) levels were 59% lower within the VAD group. When the VAC diet treatment was finished, intestinal VA (retinol) levels were restored to levels similar to the VAC group (Figure 1b). Taking into account that the intestine serves as the main tissue site of the body's retinol absorption (Underwood, 2004), the relative mRNA levels of retinoid receptors, enzymes, and binding protein carriers, including the retinoic acid receptors (RARs) and its heterodimers‐retinoic X receptor (RXRs), cellular retinol‐binding protein 2 (CRBP2), and retinoic acid‐metabolizing enzyme CYP26a1 (CYP26a1), were measured to explore the pathway of VA metabolism. In the results, the mRNA levels of RARα, RAR β, RXRα, RXRβ, CRBP2, and CYP26a1 were shown to be greatly reduced, especially CYP26a1 being lowest expressed, within intestinal tissues in the VAD group relative to the control group both in jejunum and ileum (Figure 1c).

### 3.2 The effect of VA deficiency on intestinal microbiota richness and diversity

**FIGURE 2:**
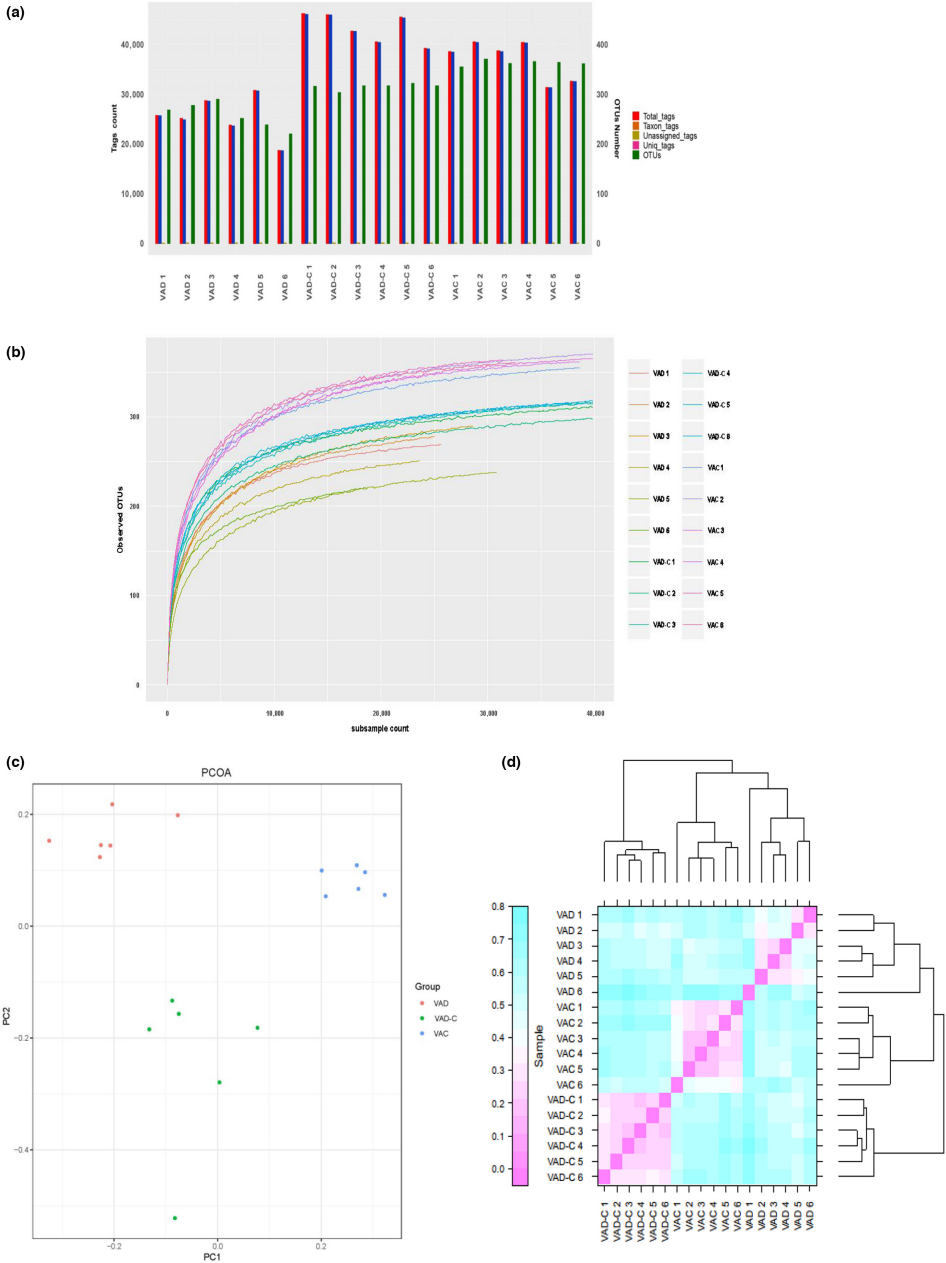
VAD alters the diversity features of community structure composition of intestinal microbiota. (a) Comparison of OTUs of mice from VAC, VAD, and VAD‐C groups. (b) Shannon–Wiener curves of mice from VAC, VAD, and VAD‐C groups. (c) PCoA of mice from VAC, VAD, and VAD‐C groups. (d) Bray–Curtis analysis of mice from VAC, VAD, and VAD‐C groups.

In all the samples, 469,359 valid sequences were obtained in total, with an average sequence length of 373 bp. Cluster comparison of OTUs with similarity greater than 97% was performed. Figure 2a shows that there were a lower number of OTUs of VAD group mice than that of the VAN group. In the samples, the Shannon–Wiener curves fitted the inference mentioned above. Even if those of the VAD group saw a relative scattering, there were generally higher Shannon curves of samples within the VAC group than the VAD group (Figure 2b). Besides, the principal coordinate analysis (PCoA) with unweight UniFrac distance was used to cluster both the differences and the similarities between microbial communities for six groups. In the unweighted Unifrac analysis, a wide variation was found among the groups. The first principal coordinate (P1), explaining 25.7% of data variance, not only separated the VAD groups but also exhibited a high similarity in the VAC and the VAD‐C groups (Figure 2c). Bray–Curtis analysis showed bacterial communities in the VAD group clustered quite differently from each other, whereas those within VAD and VAD‐C groups clustered together (Figure 2d).

### 3.3 The effect of VA deficiency on intestinal microbiota community components

**FIGURE 3:**
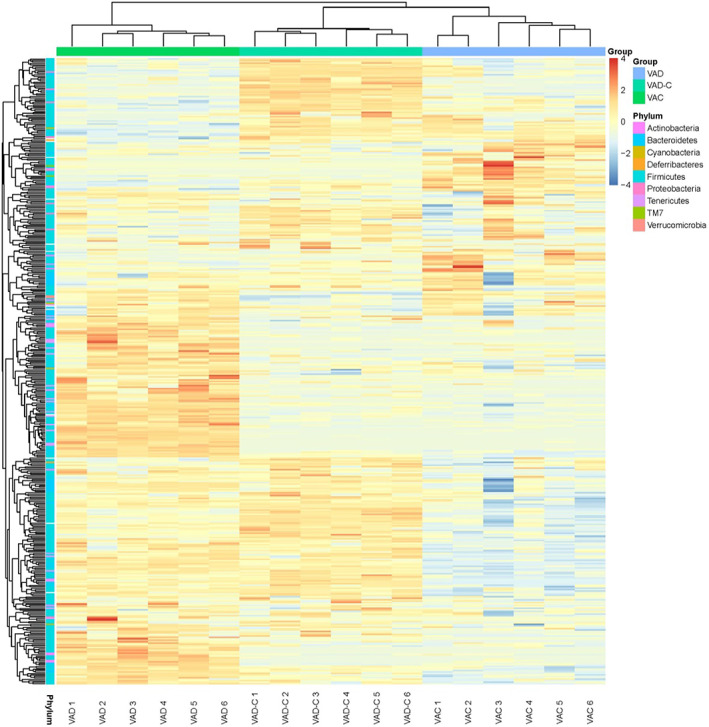
VAD alters the community cluster of bacterial communities at the phylum level. The cluster analysis for bacterial communities of mice from VAC, VAD, and VAD‐C groups.

The loading plots on the basis of the first three components were displayed as the distribution of key OTUs identified by PLS‐DA, which shows the high performance for changes differentiation and prediction within the gut microbiota. It was identified that 416 OTUs with a VIP >1.00 in total represented the key lineages for separating dominant gut microbes within each group. Figure 3 exhibits the OTUs heatmap in three groups. In the results, it was found that both these parameters and their interactions of them exerted the most influence on those 416 OTUs. In Figure 3, it could be found that the 416 OTUs in the VAD group possessed an obvious difference from those in the VAC group and the VAD‐C group by analyzing the Euclidean distance. In addition, the 416 OTUs were distributed across the phylum Actinobacteria (15 OTUs), Bacteroidetes (48 OTUs), Cyanobacteria (three OTUs), Deferribacteres (two OTUs), Firmicutes (313 OTUs), Proteobacteria (10 OTUs), Tenericutes (23 OTUs), TM7 (10 OTUs), and Verrucomicrobia (two OTUs).

### 3.4 The effect of VA deficiency on intestinal bacterial taxonomic composition at different levels

**FIGURE 4:**
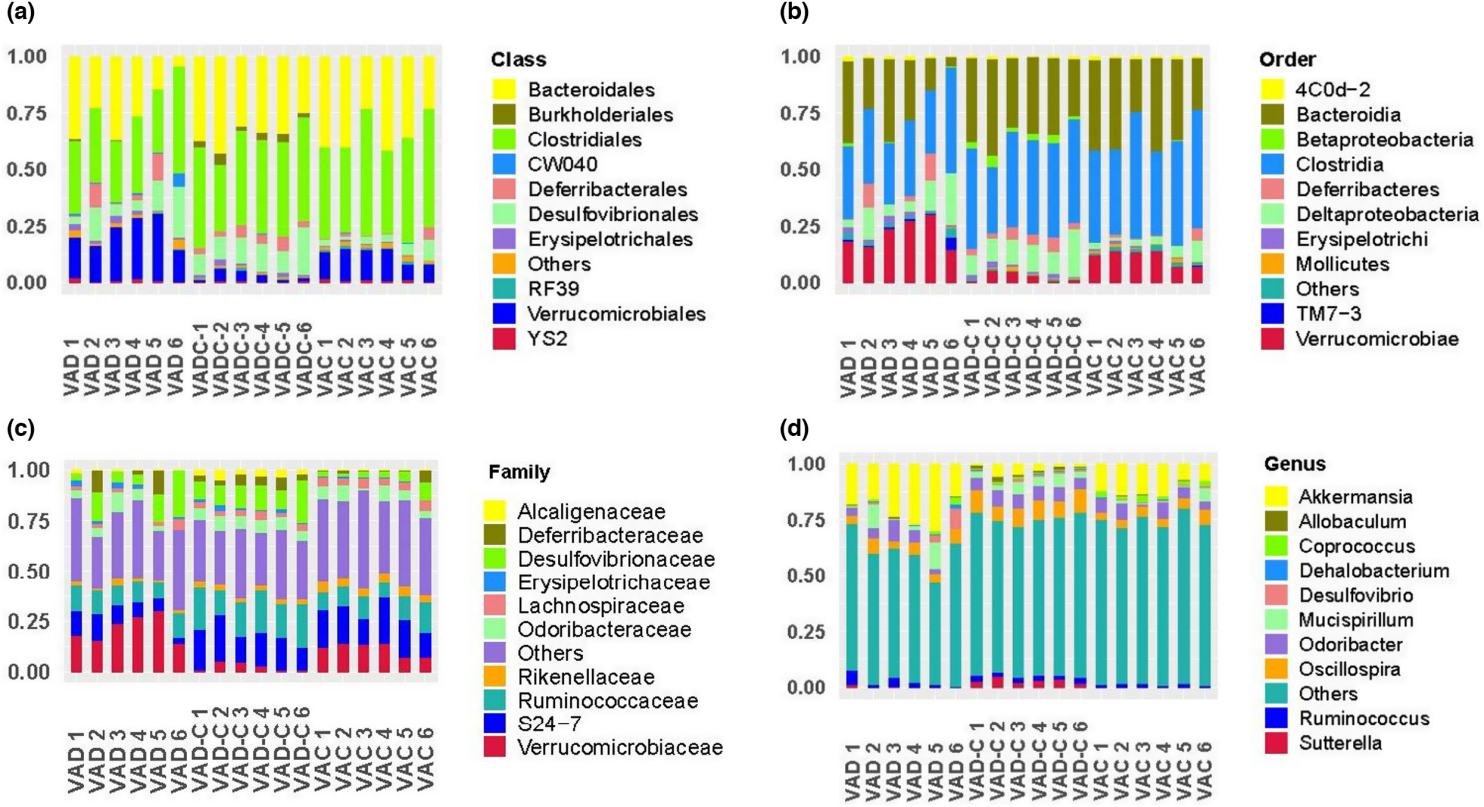
VAD caused differences in the relative abundances of the bacterial communities at different levels. The relative abundances of the bacterial communities at the class level (a), order level (b), genus level (c), and family level (d).

Figure 4 shows that the community structure of the two groups was divided by order, class, family, and genus. To be specific, at the class level, Clostridiales was the predominant class, contributing to 31.6%, 45.9%, and 43.7% of the gut microbiota within VAD, VAD‐C, and VAC groups separately. It can be seen that that was followed by Bacteroidales contributing to 20.2%, 30.4%, and 34.60% separately. As the next most dominant class, Verrucomicrobiales contributed to 4.8% of the VAD group and 20.7% of the VAD‐C group, respectively (Figure 4a). In addition, at the order level, Bacteroidia, Clostridia, and Verrucomicrobiae served as the dominant orders within the VAD group, while Bacteroidia and Clostridia were considered the prevailing order within the VAD‐C group and VAC group. Compared to the VAC group (3.99%, Figure 4b), the Verrucomicrobiae order received a high enrichment within the VAD group (20.72%). At the family level, the VAD group had more abundant Verrucomicrobiaceae (17.71%), but it is noted that the VAD group had fewer Ruminococcaceae and S24‐7 (6.9% and 9.2%, Figure 4c). What is more, at the genus level, five genera took the share of over 3% of the gut microbiota within VAD, which were the Akkermansia (27.71%), Mucispirillum (6.36%), Odoribacter (4.8%), Oscillospira (3.8%), and Ruminococcus (3.0%). However, six genera took the share of over 3% of the gut microbiota within the VAD‐C group, which were the Akkermansia (5.31%), Mucispirillum (4.1%), Odoribacter (8.5%), Oscillospira (11.9%), Ruminococcus (2.4%), and Sutterella (3.0%) (Figure 4d).

### 3.5 The effect of VA deficiency on intestinal structure and inflammation

**FIGURE 5:**
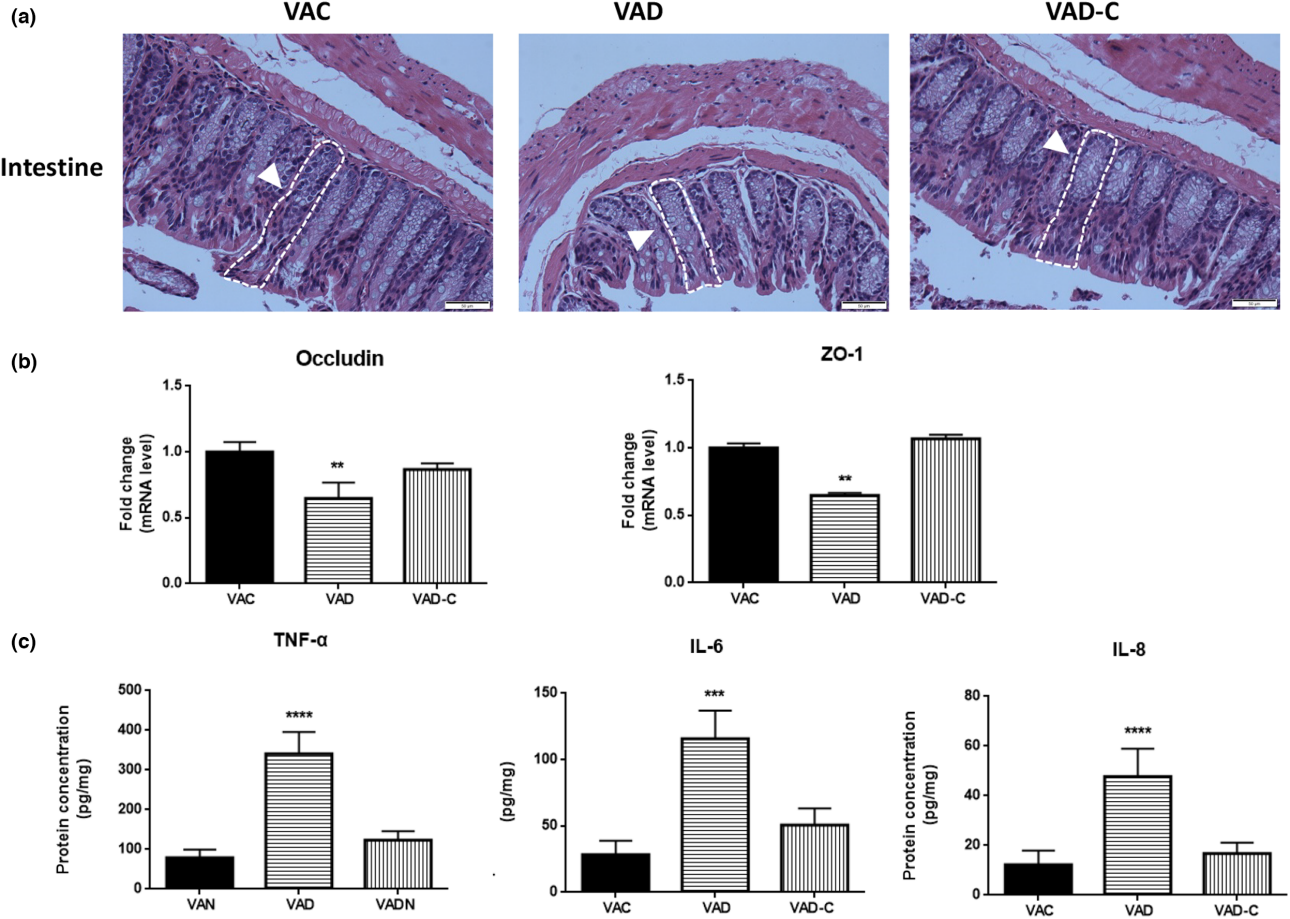
VAD destroyed and intestinal permeability and increased intestinal inflammation response. (a) Representative photomicrographs of intestinal sections of mice from VAC, VAD, and VAD‐C groups using H&E staining, and the phenotype change of intestinal tissues is indicated by white arrows. (b) PCR measurements of relative intestinal tight junction integrity markers (ZO‐1, occludin) of mice from VAC, VAD, and VAD‐C groups. (c) Intestinal local protein levels of inflammation markers (TNF‐α, IL‐6, and IL‐8) were analyzed using ELISA for mice from VAC, VAD, and VAD‐C groups. Error bars represent SE. **p < .01, ***p < .001, and ****p < .0001 in post hoc comparisons among groups after one‐way ANOVA analysis.

To study the effects of changes in the gut microbiota and influenced barrier function, we first evaluated intestinal morphological and intestinal local inflammation responses. In the intestinal morphological analysis through the use of the H&E staining, it could be found that the intestinal tissues from the VAD diet‐fed mice were partly disordered and shorter than VAC diet‐fed mice, which were found to be thin, tall, and well developed. As shown in Figure 5a, compared to those of VAC mice, crypt depth increased to 55.78 μm (1.3‐fold) in VAD mice, while pile height exhibited the opposite tendency with a 56% reduction to 165.3 μm in VAD mice. Despite no obvious difference among the three groups, VAD diet‐fed mice tend to crypt elongation (Figure 5a). In the meantime, there were lower mRNA levels of zona occludens‐1 (ZO‐1) and occludin, which served as the key markers of tight junction integrity (Brun et al., 2007), within the intestinal segment from VAD mice in comparison with those from the VAC mice (Figure 5b). Compared to those in VAC and VAD‐C mice, the protein levels of TNF‐α, IL‐6, and IL‐8 were higher within VAD mice (Figure 5c). Altogether, these multiple results support a strong relationship between gut microbiota, gut permeability, and intestinal inflammation within VAD mice.

## 4 DISCUSSION

Vitamin A and its metabolites (retinoic acid, etc.) contribute greatly to keeping a plethora of biological functions, such as metabolic and respiratory disorders in mammalian (Reboul, 2013). The tissue retinoid homeostasis is kept through the fine balance between the VA transport to the target organs and the conversion to VA ester reserves being adopted when needed. Serum VA (retinol) levels are seen to be under the tight control of the hepatic homeostatic and have no decrease till nearly depleting VA (retinol) concentration within the liver. This means that serum VA (retinol) levels just change when there is serious VA deficiency (Sommer, 2008; Underwood, 2004). In the VA‐deprived mice at 3 weeks postpartum, normal serum retinol concentrations could be found until 20 weeks (Etchamendy et al., 2003). Further, the research identified the downregulation of the serum retinal level after weaning week 16. It is speculated to be possibly caused by the serious decrease of the maternal VA level during gestation, which can bring about insufficient mother and pup. In addition, VA is needed since postnatal pups grow rapidly, possibly resulting in a further decrease in the serum retinal concentration. The small intestine serves as the first gateway for contact with dietary VA and the unique organ for its absorption and metabolism. Dietary VA enters the small intestine and is metabolized to produce retinoic acid. We further observed that the VA signal pathway, such as the retinoic transcription nuclear receptors, the carrier proteins, and the intracellular retinoid metabolic enzymes, are all reduced in the jejunum and ileum, which confirmed the sharp decrease of the tissue VA levels.

The richness, dynamics, diversity, and resilience of microbial communities keep homeostatic equilibrium and are resistant to perturbations (Das & Nair, 2019). Our data demonstrated that the sequence number and OTUs within the VAD group were significantly decreased compared to those in VAC mice. The distances of the PCoA diagram will become closer if the sample community composition becomes smaller (Avershina et al., 2013). The results showed that the group composition within VAC, VAD, and VAD‐C groups presented some obvious differences and variations within the sample community composition. As for the beta‐diversity and cluster analysis, bacterial community structure divergence between the VAD group and the VAD‐C group was disclosed. However, the VA resumption therapy restored the microbiota structure to normal levels, which can be found within the VAC group. In view of this, it can be speculated that lacking VA during the whole life cycle is likely to have an impact on the microbial microbiota composition within the intestinal mucosa.

Taking into account the community structure outcomes, the abundance of different conditional pathogenic bacteria is present with VAD mice and VAC levels at the phylum level, class level, order level, genus level, and family level, respectively. Bacteroidetes and Firmicutes fundamentally include gram‐negative bacteria, apart from gram‐positive Firmicutes microorganisms (Greenhill, 2015; Indiani et al., 2018), and some polysaccharide degrading enzymes kinds are generated by Bacteroidetes phylum, whereas there was fewer current number of microbes with polysaccharide degradation capacity within Firmicutes phylum (Allaband et al., 2019; Johnson et al., 2017). In terms of ecological imbalance, VAD brought about disproportionate abundances of Firmicutes and Bacteroidetes within the intestines, fitting other experiment researchers' results (Chen et al., 2020; Liu et al., 2016). In the above data segregation, various conditional pathogenic bacteria are shown to occur within mice with different VA levels. With the approach of exploring opportunistic pathogens to be a bioindicator, the influences of VAD in shaping the gut microbiota architecture from the class level to the genus level were explored. Our results showed that Verrucomicrobiales at the class level and Verrucomicrobiae at the order level saw an obvious decrease in the VAD group. Moreover, the order of Verrucomicrobiales, specifically Akkermansia, at the genus level was highly enriched in VAD mice. When VA administration was restored in the period of the post‐VA supplement, there was a decrease in the marked rise of Akkermansia abundance during the VAD treatment period ending. Similar to that in the Lactobacillales and Bifidobacteriales, it seems that antibiotic treatment clears a niche, where synbiotics induce Akkermansia to proliferate for a longer period. That result is likely to play a physiological role since Akkermansia serves as a mucin‐degrading bacterium residing within the gut's mucus layer (Everard et al., 2013), regulating gut metabolism and enhancing intestinal barrier integrity (Ottman et al., 2017; Raymond et al., 2016).

In various researches, the VA nutritional state was shown to be included within intestinal immunity regulation (Bai et al., 2009; Hong et al., 2014). Our results showed that intestinal permeability and intestinal inflammation both saw an increase in the response to the mice VAD and that the intestinal mechanical barrier saw the destruction. After using the VAC diet to treat the VAD diet‐fed mice, an improvement could be seen in the inflammatory response of intestinal tissues. These results were consistent with He et al.'s (2019, 2020) study, which showed that VA reverses lipopolysaccharide (LPS)‐induced intestinal barrier damage and perfects the intestinal barrier function by strengthening the tight junction proteins expression. In view of this, through bacterial population alteration, VAD is likely to damage not only immune cell number and innate immunity‐related factors expression, thereby showing independent influences of VA on intestinal mucosa‐related bacteria (Arpaia et al., 2013; Li et al., 2018). Moreover, it is also proposed that high VA concentrations may better digest and absorb nutrients, and also possess longer small intestinal lengths. In vitro study, there was inhibited differentiation of organoids that were treated with retinol and its metabolites, indicating that VA absence brought about an uncontrolled proliferation of epithelial stem cells fail to differentiate into the normal phenotype in a lot of the lining epithelia in the process of development (Wang et al., 2020). As a result, this is seen to be another mechanism for the influence of intestinal immunity within VAD mice.

To sum up, it is found that the imbalance of the intestinal microbiota that is induced by VA may promote some changes within intestinal function metabolism, especially within the intestine's capacity to keep that the inflammatory processes favored. After the lack of VA, the abundance of Akkermansia was increased, suggesting that it may be used as a potential therapeutic target.

## AUTHOR CONTRIBUTIONS

Junming Zhou and Bo Sun performed the experiments, analyzed data, and wrote the manuscript. Minli Li, Ying Feng, Haoyu Xu, Meixia Guo, and Xiaowei Wu collected tissue and analyzed the data. Xiaomin Wang conceived, designed, and directed the study.

## FUNDING INFORMATION

The work was supported by In‐Hospital Program of Jinling Hospital (To Junming Zhou, No. YGQN2021101).

## CONFLICT OF INTEREST STATEMENT

Not declared.

## Data Availability

The data that support the findings of this study are available on request from the corresponding author. The data are not publicly available due to privacy or ethical restrictions.
